# Next-Generation Sequencing Reveals Frequent Opportunities for Exposure to Hepatitis C Virus in Ghana

**DOI:** 10.1371/journal.pone.0145530

**Published:** 2015-12-18

**Authors:** Joseph C. Forbi, Jennifer E. Layden, Richard O. Phillips, Nallely Mora, Guo-liang Xia, David S. Campo, Michael A. Purdy, Zoya E. Dimitrova, Dorcas O. Owusu, Lili T. Punkova, Pavel Skums, Shirley Owusu-Ofori, Fred Stephen Sarfo, Gilberto Vaughan, Hajung Roh, Ohene K. Opare-Sem, Richard S. Cooper, Yury E. Khudyakov

**Affiliations:** 1 Molecular Epidemiology and Bioinformatics Laboratory, Division of Viral Hepatitis, Centers for Disease Control and Prevention, Atlanta, Georgia, United States of America; 2 Department of Public Health Sciences, Loyola University Chicago, Maywood, Illinois, United States of America; 3 Department of Medicine, Loyola University Chicago, Stritch School of Medicine, Maywood, IL, United States of America; 4 Komfo Anokye Teaching Hospital, Kumasi, Ghana, West Africa; 5 Kwame Nkrumah University of Science and Technology, Kumasi, Ghana, West Africa; University of Cincinnati College of Medicine, UNITED STATES

## Abstract

Globally, hepatitis C Virus (HCV) infection is responsible for a large proportion of persons with liver disease, including cancer. The infection is highly prevalent in sub-Saharan Africa. West Africa was identified as a geographic origin of two HCV genotypes. However, little is known about the genetic composition of HCV populations in many countries of the region. Using conventional and next-generation sequencing (NGS), we identified and genetically characterized 65 HCV strains circulating among HCV-positive blood donors in Kumasi, Ghana. Phylogenetic analysis using consensus sequences derived from 3 genomic regions of the HCV genome, 5'-untranslated region, hypervariable region 1 (HVR1) and NS5B gene, consistently classified the HCV variants (n = 65) into genotypes 1 (HCV-1, 15%) and genotype 2 (HCV-2, 85%). The Ghanaian and West African HCV-2 NS5B sequences were found completely intermixed in the phylogenetic tree, indicating a substantial genetic heterogeneity of HCV-2 in Ghana. Analysis of HVR1 sequences from intra-host HCV variants obtained by NGS showed that three donors were infected with >1 HCV strain, including infections with 2 genotypes. Two other donors share an HCV strain, indicating HCV transmission between them. The HCV-2 strain sampled from one donor was replaced with another HCV-2 strain after only 2 months of observation, indicating rapid strain switching. Bayesian analysis estimated that the HCV-2 strains in Ghana were expanding since the 16^th^ century. The blood donors in Kumasi, Ghana, are infected with a very heterogeneous HCV population of HCV-1 and HCV-2, with HCV-2 being prevalent. The detection of three cases of co- or super-infections and transmission linkage between 2 cases suggests frequent opportunities for HCV exposure among the blood donors and is consistent with the reported high HCV prevalence. The conditions for effective HCV-2 transmission existed for ~ 3–4 centuries, indicating a long epidemic history of HCV-2 in Ghana.

## Introduction

Hepatitis C virus (HCV) infection is a major global health issue. Globally, >185 million people are estimated to be infected with HCV and ∼350,000 people die every year from HCV-related liver diseases. [1,2] The severity of the disease varies from asymptomatic infection to cirrhosis and hepatocellular carcinoma [3]. Most HCV infections remain asymptomatic for years. New therapies with high rates of sustained virologic response or cure [4,5] are impractical owing to exorbitant cost for the majority of HCV-infected persons in sub-Saharan Africa, where the infection is highly prevalent [6]. In Ghana, the prevalence of infection among blood donors exceeds endemic levels with prevalence of up to 11.6% among male donors [7,8]. The national demand in Ghana for safe blood for patients is more than 250,000 units annually, but the proportion collected is less than 50% [9]. Rejection of blood due to infection with HCV contributes to this shortage [8,10,11].

The high prevalence of HCV infection among blood donors suggests that mechanisms responsible for HCV transmission in this population in Ghana are sustainable and efficient. Exposures to infected blood and blood products through unsafe injection practices, transfusion of untested blood, unsterile medical and dental procedures, and traditional medical and cosmetic procedures such as scarification are major risk factors for HCV transmission[12,13]. Recently, we reported that traditional circumcision, home birth, tribal scarring and HBV co-infection are major risk factors associated with HCV infection among blood donors in Kumasi, Ghana [14].

Mathematical models of viral kinetics estimate that more than 10^12^ virions are produced per day in each HCV-infected person [15]. The rapid replication and lack of RNA-polymerase proofreading result in the mutation rate of (1.15±0.29)×10^−4^ per nucleotide per replication round [16], which is within the range of other RNA viruses [17]. Consequently, HCV is very heterogeneous and can be classified into 7 major genotypes and >70 subtypes [18]. Many distinct but highly related HCV variants coexist in each infected individual. Hypervariable region 1 (HVR1) located at the N-terminus of the E2 gene is one of the most heterogeneous regions of the HCV genome and used frequently to assess intra-host HCV heterogeneity [19–22].

The aims of the present study were to characterize and investigate the HCV genetic diversity in a population of blood donors in Ghana where the high prevalence and impact of HCV infection on public health are increasingly recognized. Using conventional sequencing from three HCV genomic regions, we show that blood donors in Kumasi-Ghana are infected with a very heterogeneous HCV population of HCV-1 and HCV-2, with HCV-2 being prevalent. The data obtained using the next-generation sequencing (NGS) of HVR1 point to frequent opportunities for HCV exposure among the blood donors. It seems that the conditions for the effective HCV-2 transmission have existed for ~ 3–4 centuries in Ghana.

## Materials and Methods

### Study subjects and ethics statement

Serum samples (n = 363) were obtained from a random population of recalled male blood donors from the Komfo Anokye Teaching Hospital (KATH) blood bank, Kumasi Ghana, West Africa collected in 2013/2014 [14] and tested in this study. All donors were hepatitis C treatment-naïve. The study population was derived from individuals who had previously donated blood at the KATH in Kumasi, Ghana for the last five years. When individuals donate blood, screening is performed for HCV antibody and a database of the results is kept. Individuals seropositive for anti-HCV were recalled. Seronegative donors who had donated at the time closest to each HCV-positive screen-case were also recalled to obtain HCV-negative controls. The samples for this study were delinked from personal identifiers. The samples were initially tested for HCV infection using the NS5b region encompassing positions 8275–8616 as previously described [23,24]. Only samples that were NS5b positives (n = 65) were further analyzed. [14,25] The study was approved by the ethics committees in KATH-Kumasi, Ghana, and the Institutional Review Board for the protection of human subjects at Loyola University Medical Center, Maywood, IL, and the Centers for Disease Control and Prevention, Atlanta, GA, to test anonymized, unlinked samples for HCV. Written informed consent was obtained from all subjects. In all, this study was conducted in accordance with principles in the Declaration of Helsinki for medical research on human subjects.

### RNA extraction, cDNA synthesis and PCR

Total nucleic acid was extracted from all plasma samples using the automated Roche MagNA Pure LC Instrument and the MagNA Pure LC Total Nucleic Acid Isolation kit (Roche Diagnostics, Indianapolis, IN), and eluted. cDNA was generated using the high-temperature capability SuperScript® VILO™ cDNA Synthesis Kit (Invitrogen, Life Technologies, Carisbad, CA) on an ABI PRISM® 9700 PCR system. The reverse transcription PCR conditions were as follows: 25°C for 10 min, 42°C for 90 min, and 85°C at 5 min. Amplification of the NS5b-gene region was done as previously described [23,24] using the LightCycler® 480 software (Version 1.5.0.SP3, Roche, Indianapolis, IN). Samples that were found to be NS5b positive were subjected to 5'UTR and HVR1 amplification by a nested PCR targeting the 5'UTR and the E1/E2 junction region which contains the HVR1 region, respectively, using specific primers and thermal cycling conditions as previously described in detail [26,27].

### DNA sequencing

Amplicons derived from the PCR amplifications were purified (Millipore multiscreen PCR filter plate, Billerica, MA) and sequenced using their respective nested primers and the BigDye v3.1 chemistry sequencing kit (Applied Biosystems) by an automated sequencer (ABI 3130xl, Applied Biosystems, Foster City, CA) as previously described [23,24]. HCV genotypes were classified based on the NS5b, 5'UTR and consensus HVR1 sequences as described [23,24,26,27].

We sequenced the intra-host HCV HVR1 variants from all HCV-positive donors using the 454/Roche GS Junior system. The HVR1 region of the HCV genome was amplified independently with fusion primers including the 454-primer key (A and B for forward and reverse primers, respectively), with a different multiple identifier (MID) for each sample, and an HCV-specific sequence as recently described [23]. The PCR products were purified using the 2% size-select gel (Invitrogen), and quantified with the Agilent 2200 TapeStation system (Agilent Technologies, Inc., Waldbronn, BW, Germany, or Santa Clara, CA). To analyze pyrosequencing data, original sequence reads (raw data) were processed using SFFFILE tools. Sequence reads belonging to each sample were identified and separated using MIDs [23]. Low quality reads were removed. The pyrosequencing data were processed with the KEC error correction algorithm for rapid recovery of high-quality haplotypes from reads obtained by deep pyrosequencing of amplicons from heterogeneous viruses such as HCV [28]. KEC is based on the analysis of a distribution of observed k-mers (sub-strings of reads of a fixed length k). An earlier validation showed that the KEC imparts very high accuracy to identifying true haplotypes and estimating their frequencies [28]. In the context of this study, haplotypes are defined as different sequence variants found within a sample. Each haplotype has an associated frequency, which corresponds to the number of NGS cleaned reads matching the haplotype. So, each sample contains a set of haplotypes with different frequencies and the major haplotype has the highest observed frequency.

### Genetic analysis

Consensus sequences obtained by Sanger sequencing were initially assembled and cleaned using SeqMan and MegAlign programs from the Lasergene DNA and Protein analysis software (version 10.1.2, DNASTAR Inc., Madison, WI). All sequences were aligned using Geneious Pro version 5.5.8 created by Biomatters, NewZealand, and also with MUSCLE as implemented in Mega5 [29]. Phylogenetic trees were inferred for each dataset using the maximum likelihood method from the MEGA5 software [29]. Genotypes were classified by comparing sequences obtained in this study with representative GenBank sequences following which reference sequences were removed. Analyses of minimum distances, maximum distances, nucleotide diversity, frequency of major haplotypes and calculations for sequence relatedness were performed on the HVR1 quasispecies using statistical and bioinformatics toolboxes implemented in MATLAB software package version 2011B (The MathWorks, Inc., Natick, MA). The program ARLEQUIN [30] was used for the comparison of paired populations by calculating the fixation index (Fst) and pairwise level of significance between time points for samples collected at different time-points according to Weir and Cockerham [31]. The Fst is a measure of population differentiation due to genetic structure. The fixation index can range from 0 to 1, where 0 means complete sharing of genetic material and 1 means no sharing. For values equal to 1 (meaning no sharing), the populations are described as fixed. If populations are referred to as fixed, it means that they do not share any sequence/haplotype with one another. Significance level was set as below 0.05.

### Pathfinder networks (PFnet)

In order to visualize the genetic distances among all sequences of a single patient at different time-points, we used the PFnet method [32]. PFnet was originally developed by cognitive scientists and is used in the study of knowledge discovery, citation patterns, information retrieval, and data visualization [33]. The goal of PFnet is to remove links when they are not on shortest paths [33]. PFnets have the ability to derive more accurate local structures than other comparable algorithms, such as multidimensional scaling and minimum spanning trees, for which the relationships identified between neighboring points are often significantly different from the original data. In addition, the PFnet represents the most "salient" relationships present in the data and its topological analysis reveals patterns in the data that lead to fruitful interpretations of its underlying organization [34]. The network generation procedure incorporates two parameters: (1) the r parameter defines the metric used for computing the distance of paths. The r parameter is a real number between 1 and infinity; and (2) the q parameter constrains the number of indirect proximities examined in generating the network. The q parameter is an integer between 2 and n-1, where n is the number of nodes or items. A network generated with particular values of q and r is called a PFnet (q,r). Both of the parameters have the effect of decreasing the number of links in the network as their values are increased. The network with the minimum number of links is obtained when q = n-1 and r = ∞, i.e., PFnet(n-1,∞). In general, the PFnet(n-1,∞) includes all of the links in any minimum spanning tree. In this study we first calculated the Hamming distances among all sequences and then calculated the PFnet(n-1,∞) with Matlab [35].

### Molecular clock analysis

The evolutionary history of HCV-2 was reconstructed using the Bayesian coalescent approach as implemented in BEAST (ver. 1.6.0) [36]. The date of the most recent common ancestor (MRCA) of HCV-2 strains from Ghana were calculated using 300 nt NS5b sequences. Divergent time and skyline plot was performed using the constant size, exponential and expansion growth priors, a strict clock, a substitution rate of 5×10^−4^ nucleotide substitutions per site per year, and a piecewise-constant skyline model as we previously described [24,37]. The convergence of parameters was inspected using Tracer version 1.5, with uncertainties depicted as 95% highest probability density (HPD) intervals. This process was not applied to HCV-1 due to the limited number of samples which potentially increases the likelihood of error estimation.

### GenBank numbers

Sequences reported in this study have been submitted to GenBank under accession numbers KR233026-KR233155.

## Results

### Phylogenetic analysis

**Fig 1:**
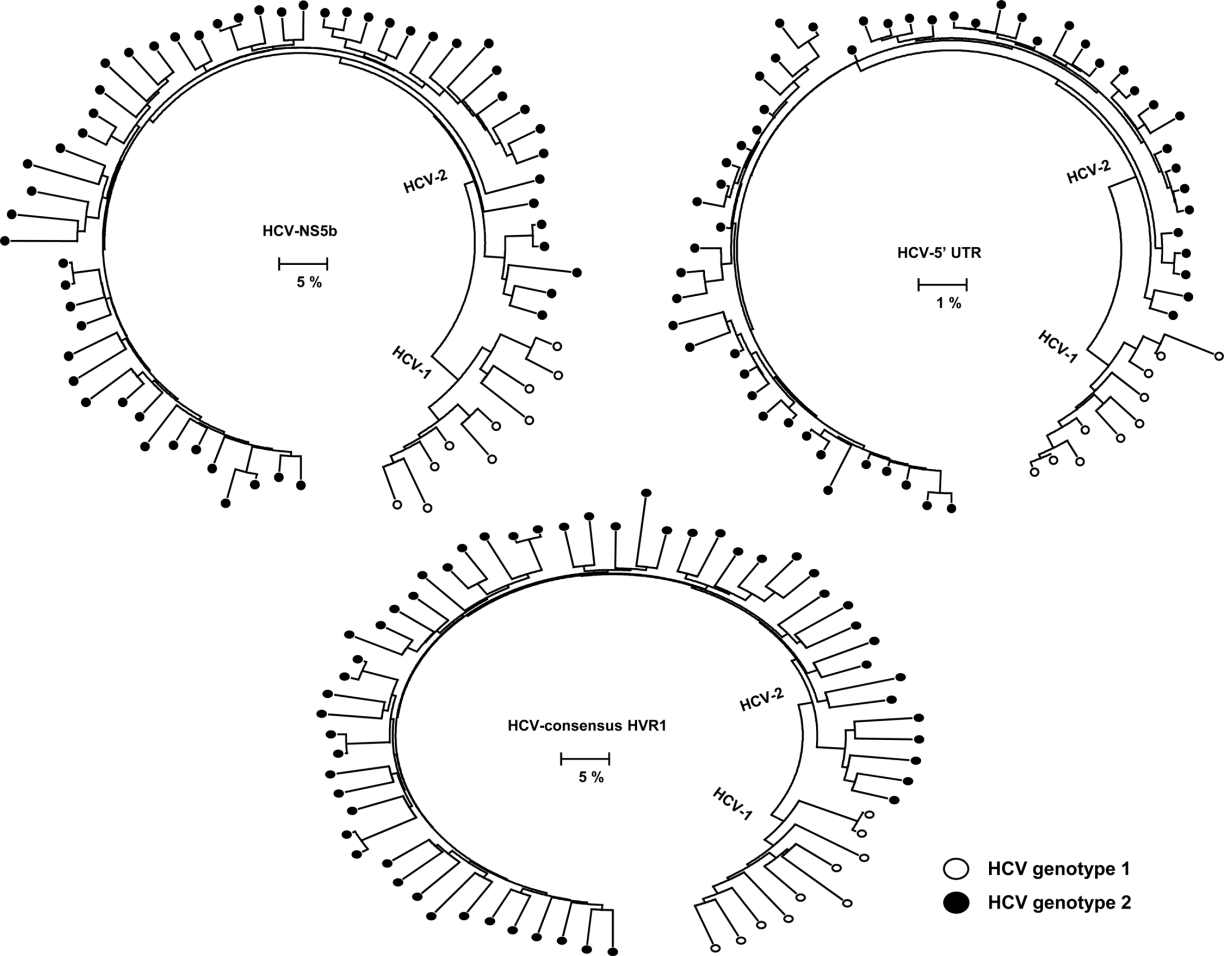
Maximum likelihood tree reconstructed with consensus NS5b, 5'UTR and consensus HVR1 gene sequences generated in this study. HCV-1 isolates are shown in black unfilled circles while HCV-2 are shown in black filled circles.

**Fig 2:**
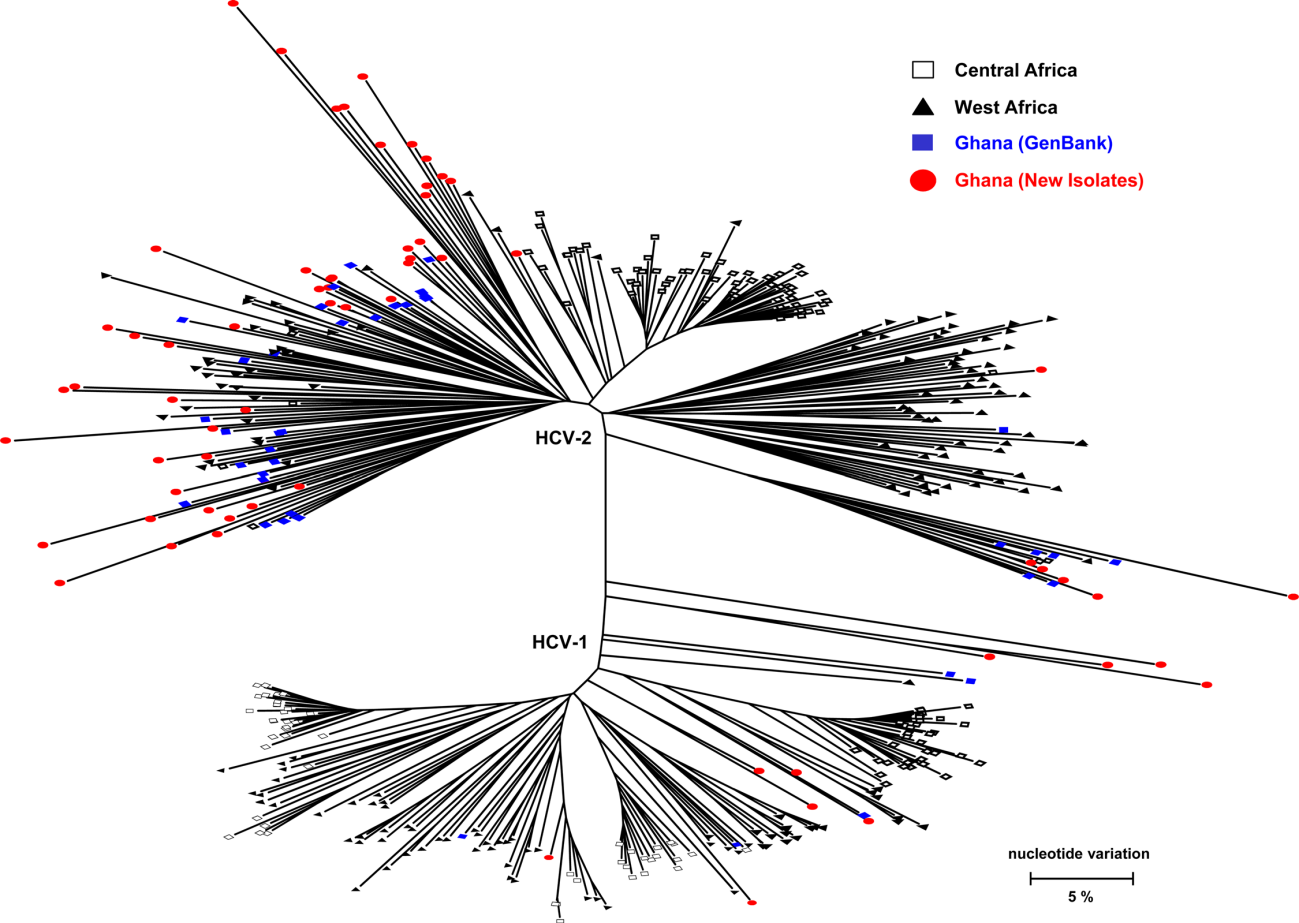
Maximum likelihood tree reconstructed with NS5b gene sequences from West and Central Africa. Sequences from Ghana generated in this study are shown as red filled circles, those reported from West Africa as black filled triangles and those from Central Africa as black unfilled squares. Ghanaian sequences obtained from the GenBank are shown in blue filled circles.

Among the 363 blood donors studied, 65 were HCV-RNA positive. Phylogenetic analysis of the NS5b sequences showed that all isolates belong to two major clusters, representing HCV genotype 1 (HCV-1; n = 10; 15.4%) and genotype 2 (HCV-2; n = 55; 84.6%). Analysis of the 5'UTR and consensus HVR1 sequences confirmed classification into the two genotypes (Fig 1). There is no genotype misclassification based on the genomic fragments used, indicating that there was no inter-genotype recombination. The NS5B sequences from Ghana and other West African countries are intermixed in the phylogenetic tree. While the NS5B sequences from Central Africa tend to be organized into tight clusters, sequences from Ghana are represented with long tips scattered across the phylogenetic tree (Fig 2).

### Intra-host diversity

**Fig 3:**
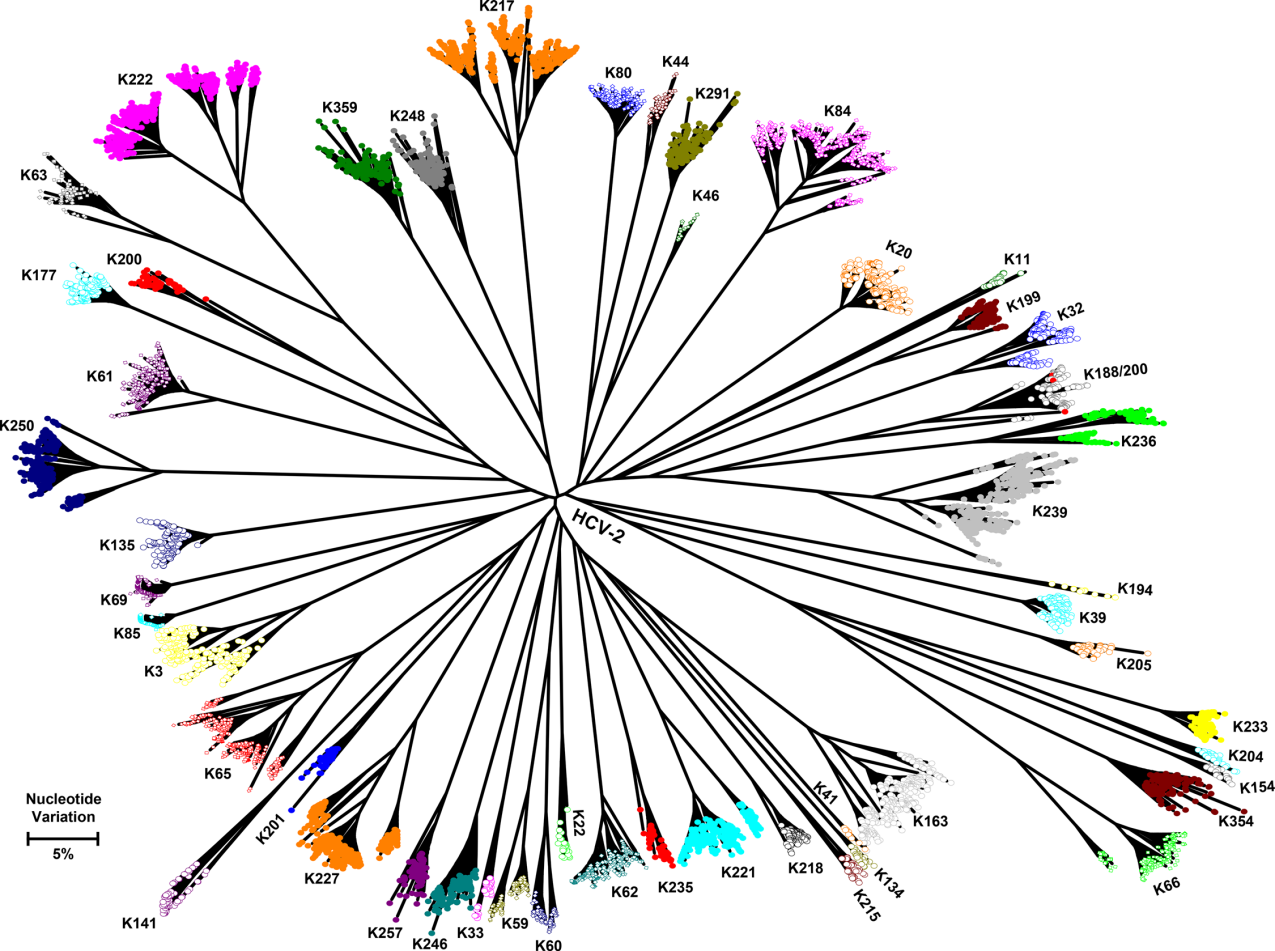
Maximum likelihood tree reconstructed with intra-host variants of HVR1 gene sequences of HCV genotype 2. Only unique haplotypes are shown. Individual samples are labeled with distinct identifiers beginning with letter ‘k’ and displayed with different colors.

**Fig 4:**
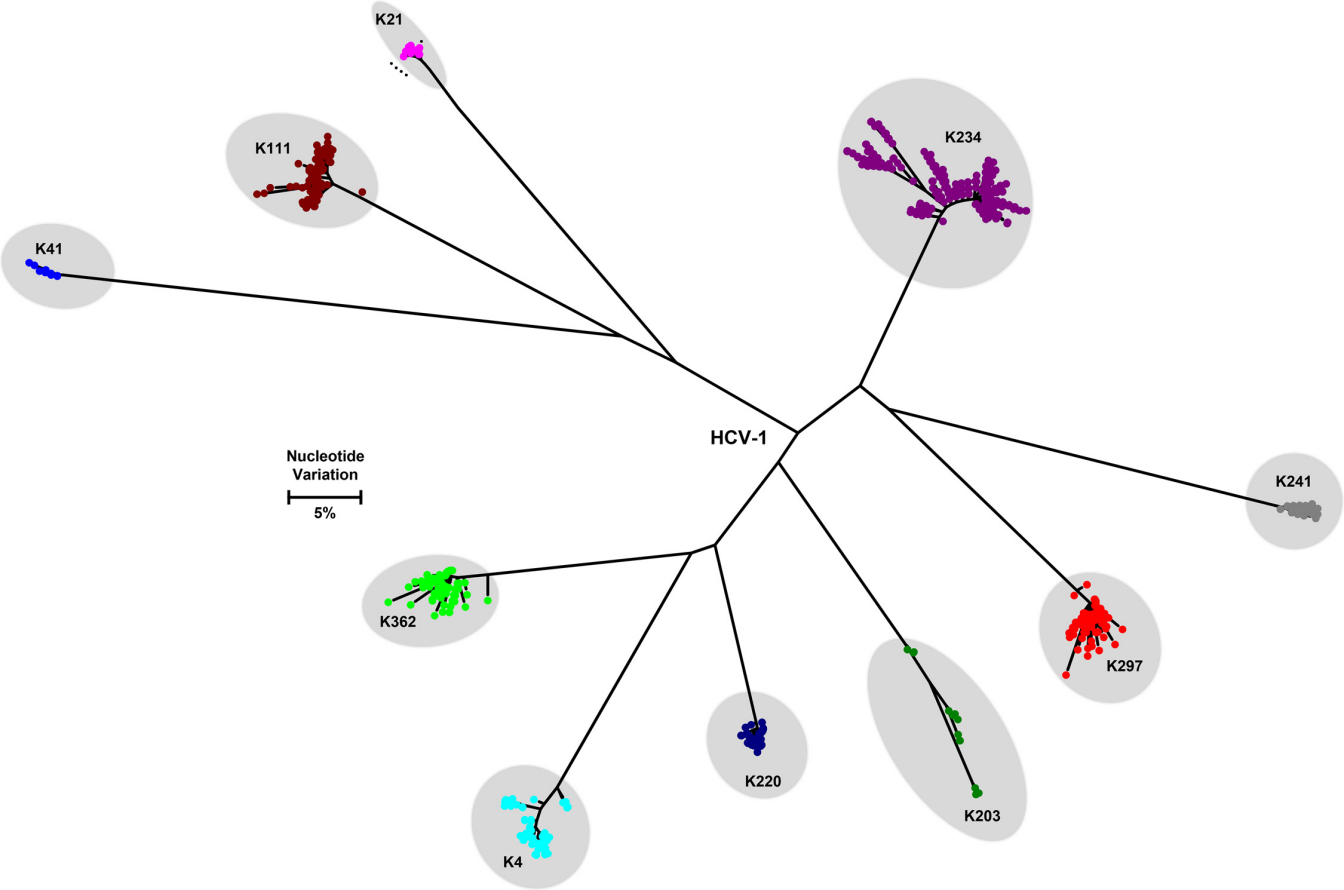
Maximum likelihood tree reconstructed with intra-host variants of HVR1 gene sequences of HCV genotype 1. Only unique haplotypes are shown. Individual samples are labeled with distinct identifiers beginning with letter ‘k’ and displayed with different colors.

Intra-host HCV HVR1 variants were sequenced using the GS Junior system from 9 individuals infected with HCV-1 and 51 infected with HCV-2. Only 5 HCV strains (K26, K254, K270, K336 belonging to HCV-2 and K368 belonging to HCV-1) were not evaluated. Donor K41 is infected with 2 HCV strains, one belonging to HCV-1 and the other to HCV-2 (Figs 3 and 4). The nucleotide diversity of HCV-1 strains range from 0.1% to 2.4% (Mean 0.9%) and of HCV-2 strains from 0.03% to 5.9% (Mean 3.8%). The frequency of the major haplotype for HCV-1 and HCV-2 strains varies between 11.7%-82.9% and 3.2%-93%, respectively. The maximum distances among intra-host HCV variants in each patient range from 1.7% - 15.2%, with the distances varying from 1.7% - 10.2% and 2.3% - 15.2% for HCV-1 and HCV-2 strains, respectively. Although the analyses seem to indicate a greater intra-host diversity of HCV-2 than HCV-1 strains, this difference is statistically insignificant.

The intra-host maximum-distance distribution overlap with the distribution of the inter-host minimum distances. For example, the minimum distance between the K59 and K60 HCV-2 strains is 14.2%, which is within the range of the maximum intra-host distances of HCV-2 strains (2.3%-15.2%).

### Variant sharing between 2 donors

Donor K200 (28 years old male) is infected with 2 HCV-2 strains (Fig 3). Intra-host variants of one of the strains are intermixed with variants from HCV-2 identified in donor K188 (38 years old male). The minimum distance between intra-host HCV variants from K188 and K200 is 0.33%. This is way below a minimal hamming distance for the relatedness threshold (3.7%) that we have recently stablished using a set of 32 epidemiologically confirmed outbreaks [38]. These findings indicate that these 2 patients share three HCV-2 strain, and, thus, are linked by transmission. Considering that only a few intra-host variants of the HCV-2 strain shared by these 2 donors were sampled from K200, who is also infected with another HCV-2 strain (Fig 3), K188 may be the source of the shared HCV-2 strain. Typically, the transmitting host (K188; nucleotide diversity = 2.2%) has greater diversity compared to the recipient’s diversity (K200; nucleotide diversity = 1.7%). Although genetic data suggest HCV transmission between these 2 donors, epidemiological investigation did not reveal any known risk factors, besides being blood donors.

### Intra-host evolution

**Fig 5:**
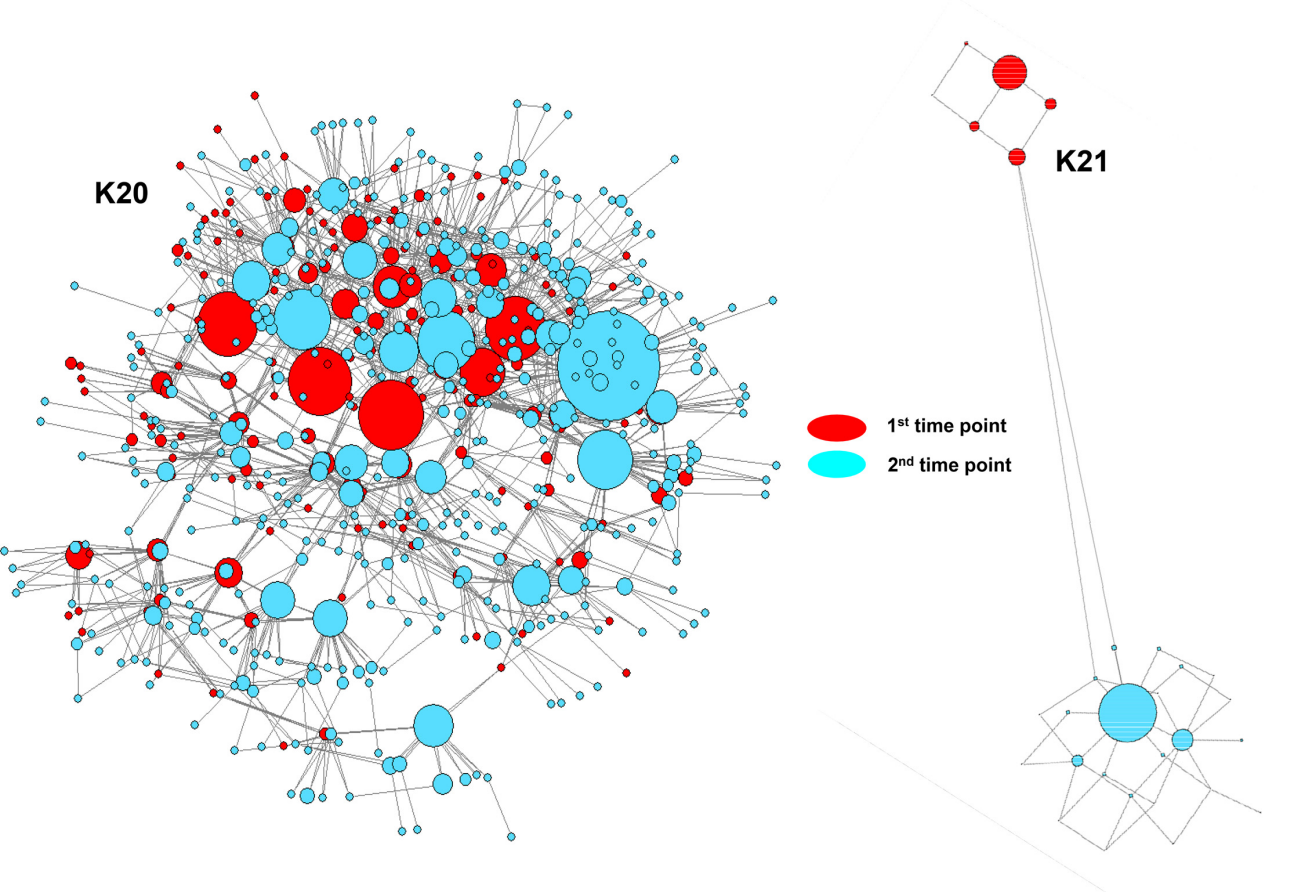
PFnet of all sequences present in two patients at different time points. Each time point is shown with a different color. Sequences found on the first time point are shown in red and the second time point in blue. Each node represents a single sequence variant. The size of the node reflects frequency of the corresponding variant in the population. This network includes all of the links in any minimum spanning tree. The time interval between each time point is ~2 months.

Samples from donors K20 and K21 were collected twice ~2 months apart from each other (Fig 5). For K20, the minimal distance between HCV variants sampled from the first and second time-points is ~0.001%. The intra-host genetic diversities do not change significantly, being 1.4% and 1.5% at the first and second time-point, respectively. The frequency of sampling of the major variant is 11.8% and 12.2% for the first and second time-points, respectively. For K21, however, the minimal distance between the first and second time-points is ~5.9%. The intra-host diversity and the frequency of sampling of the major variant increase after ~2 months from 1.9% to 2.9%, and from 67% to 81%, respectively. Analysis of pairwise distances among intra-host HCV variants between time-points shows a statistically significant difference between intra-host populations from the first and second time-points (p = 0.0001; fixation index = 0.98) for K21. However, for K20, the difference between intra-host HCV populations from 2 time-points is not statistically significant.

### Epidemic history

**Fig 6:**
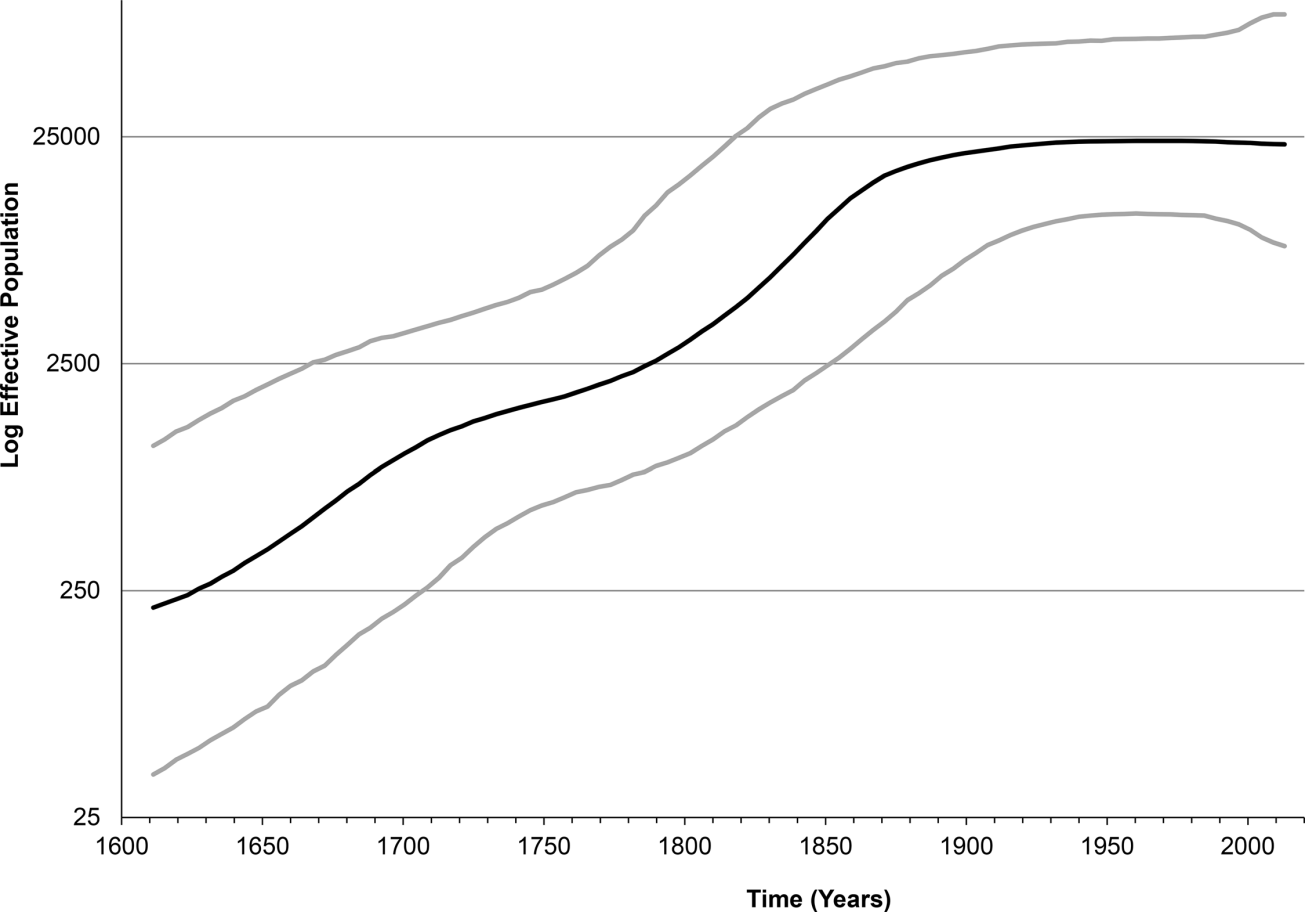
Bayesian skyline plot, showing the epidemic history of HCV genotype 2. The thick black line in the middle represents the estimated mean effective number of infections through time in years. The two grey lines represent the 95% highest posterior density of this estimate.

Using the NS5B sequences, the MRCA for the sampled HCV-2 strains is estimated to have existed ~497 years ago (95% HPD: 402–617 years). Bayesian skyline plot reveals a continuous epidemic growth until the end of 19th century, with the epidemic stabilizing in 20th century (Fig 6).

## Discussion

In this study, 65 blood donors residing in Kumasi, Ghana were found to be infected with a very heterogeneous population of HCV strains. Using sequences of three HCV genomic regions, 5'UTR, HVR1 and NS5B, revealed that all HCV variants belonged to two genotypes, HCV-1 and HCV-2, with HCV-2 infecting 85% of the tested donors. This finding is consistent with previous studies reporting that HCV-2 is more prevalent than HCV-1 in Ghana [39,40]. When the available HCV variants sampled from West Africa were analyzed, HCV-1 and HCV-2 variants from central Africa were found to form tight clusters while the HCV variants from Ghana and other West African countries were found intermixed (Fig 2). Lack of country-specific clusters and dispersion of the Ghanaian HCV variants across the entire HCV-2 in the phylogenetic tree suggest that the sample of HCV NS5B sequences in this study was obtained from a very large and heterogeneous HCV-2 population that has been circulating in Ghana [41].

In addition to the substantial inter-host HCV diversity, NGS analysis shows a very extensive heterogeneity of intra-host HCV populations. The most intriguing finding from this analysis is the frequency, with which these donors have had opportunities to be infected with more than one HCV strain. For example, donor K41 is infected with HCV-1 and HCV-2 strains (Figs 3 and 4). Donor K200 has an intra-host HCV population composed of variants that belong to two different HCV-2 strains, with one of the strains being shared with donor K188, which suggests a transmission linkage between K188 and K200 (Fig 3). Only three variants are shared, i.e., limited variants effectively establish infection similar to what is observed in HIV-1 [42,43]. Both donors have no known risk factors for HCV infection with the exception of being repeat blood donors. By chance, donors K20 and K21 were tested twice over ~2 months. The intra-host HCV population remained stable in K20, whereas it changed significantly in K21 (Fig 5). The genetic distance between HVR1 sequences from the two time-points is >5.9%, indicating that the HCV variants sampled from K21 only two months apart belong to two different HCV-2 strains. The mechanism of the switch from one HCV strain to another in this donor is not known. It is possible that K21 is infected with two closely related but genetically distinct HCV-2 strains, which vary in frequency during infection. Such fluctuations among intra-host HCV subpopulations over time were recently described [27,44]. Also, K21 could have been super-infected with a second HCV strain in the period between two donations. In general, it is striking to find three among 65 donors tested to be infected with more than one HCV strain. This finding indicates frequent opportunities for the donors in Kumasi to be exposed to HCV.

Analysis of NGS data shows variable structure of intra-host HCV populations as reflected by a significant variation of frequency of the major variants in different donors. This finding most probably indicates the detection of HCV at different stages of infection and under different selection constraints resulting in selective sweeps, background selections, positive or negative selection of varying strengths. Many HCV strains in the donors are composed of several distinct subpopulations of intra-host variants (Figs 3 and 4), which results in a very broad intra-host genetic heterogeneity. The maximum genetic distances among intra-host variants overlap with minimal genetic distances between HCV strains. These observations suggest that, at least, some of the donors had been infected for a long time and developed chronic HCV infection, which is often associated with the increase in intra-host HCV heterogeneity [27,44,45]. As the donors were predominantly young [14] and considering their narrow age distribution, they were most probably infected with HCV at different stages of their life from birth to early adulthood. The existence of numerous genetically distant subpopulations of intra-host HCV variants in many donors indicates infections with multiple founders. This finding supports the earlier observation of establishment of HCV infections from >1 founder [46]. Also, it may indicate: (1) propensity of HCV-2 to extensive intra-host genetic diversity; (2) a frequent re-infection in the past with closely related HCV strains, resulting in formation of current strains from co-evolving closely related HCV-2 strains; or (3) a long evolution of each strain in the human population, leading to extensive intra-host heterogeneity.

Indeed, HCV-2 infections have a long history in Ghana. The MRCA for all HCV-2 strains sampled in this study was estimated to have existed ~500 years ago. However, it should be noted that analysis of sequences linked to a limited duration of sample collection could result in estimating tMRCA inaccurately. The observations of long tips and lack of tight clustering in the phylogenetic tree for the Ghanaian sequences (Fig 2) indicate a significant variety of HCV-2 strains circulating in a single locale in Ghana, which may be explained by either a single massive introduction of HCV strains or introduction of numerous viral lineages over a long period of time. Alternatively, this observation suggests that Ghana is, or a part of, the geographic origin of HCV-2, which is consistent with the earlier hypothesis on the origin of this genotype in West Africa [39,41].

The skyline plot analysis shows that the effective number of HCV-2 infections grew continuously until the end of 19^th^ century (Fig 6), thus highlighting persistence of condition conducive to effective transmission of this genotype in Ghana since its origin. The skyline plot levels off during the entire 20^th^ century, which possibly suggests a decline in the rate of transmissions in the geographic region. However, this suggestion is contrary to population expansions detected for HBV and HCV in West and Central Africa in the 20^th^ century [24,47,48], thought to be associated with smallpox vaccination programs and other public health interventions in the region. Additionally, the long tips for the HCV sequences from Ghana in the phylogenetic tree (Fig 2) show that the evolutionary history of many recent substitutions is not adequately accounted for, thus prompting a certain possibility that a more extensive sampling from the Ghanaian HCV population may result in a more accurate assessment of the HCV-2 history in the 20^th^ century.

In conclusion, the blood donors in Kumasi-Ghana, are infected with a very heterogeneous HCV population that is composed of two HCV genotypes, with HCV-2 being prevalent. The detection of three cases of infections with more than one HCV strain and one case of transmission suggests frequent opportunities for HCV exposure among the blood donors and is consistent with the reported high HCV prevalence in this population in the country [7,8,14]. The data obtained here indicate that the conditions for the effective HCV-2 transmission existed for several centuries. Collectively, all these findings point to a very long and complex epidemic history of HCV-2 in Ghana.
